# Antifreeze protein complements cryoprotective dehydration in the freeze-avoiding springtail *Megaphorura arctica*

**DOI:** 10.1038/s41598-020-60060-z

**Published:** 2020-02-20

**Authors:** Laurie A. Graham, Marie E. Boddington, Martin Holmstrup, Peter L. Davies

**Affiliations:** 1 Department of Biomedical and Molecular Sciences, Queen’s University, Kingston, ON Canada; 2 Section of Terrestrial Ecology, Department of Bioscience, Aarhus University, Vejlsøvej 25, 8600 Silkeborg, Denmark; 3 Arctic Research Center, Aarhus University, Ny Munkegade 114, 8000 Aarhus C, Denmark

**Keywords:** Biochemistry, Evolution, Molecular biology

## Abstract

The springtail, *Megaphorura arctica*, is freeze-avoiding and survives sub-zero temperatures by cryoprotective dehydration. At the onset of dehydration there is some supercooling of body fluids, and the danger of inoculative freezing, which would be lethal. To see if the springtails are protected by antifreeze proteins in this pre-equilibrium phase, we examined extracts from cold-acclimated *M. arctica* and recorded over 3 °C of freezing point depression. Proteins responsible for this antifreeze activity were isolated by ice affinity. They comprise isoforms ranging from 6.5 to 16.9 kDa, with an amino acid composition dominated by glycine (>35 mol%). Tryptic peptide sequences were used to identify the mRNA sequence coding for the smallest isoform. This antifreeze protein sequence has high similarity to one characterized in *Hypogastrura harveyi*, from a different springtail order. If these two antifreeze proteins are true homologs, we suggest their origin dates back to the Permian glaciations some 300 million years ago.

## Introduction

The springtail, *Megaphorura arctica* Tullberg 1876, is widely distributed in the northern parts of the Palaearctic region where it is common and abundant along sea shores^1^. Aggregations of the species are often found in decaying seaweed of the supra-littoral zone, under stones and in moss below bird colonies^2,3^. Measurements of the winter temperature of its habitat show that the species in the field is able to survive temperatures of −20 °C or lower^4^. Freeze-tolerance (i.e. tolerance of organismal ice) has never been observed in springtails, and it is therefore generally accepted that they belong to the freeze-avoiding species^5,6^. As the name of this overwintering strategy indicates, these species have physiological adaptations enabling them to avoid internal ice formation even though the ambient temperature for long periods can be much lower than the melting point of their hemolymph. However, springtail species have followed two fundamentally different trajectories of adaptation to subzero survival by freeze-avoidance. Species living predominantly on the ground surface or in vegetation (epigeic species) have relatively impermeable cuticula and are typical freeze-avoiders with high capacity for supercooling similar to many insects^6,7^. Other springtails inhabiting deeper layers of the soil (hemi- and eu-edaphic species), have little cuticular resistance to desiccation, and base their freeze-avoiding capacity on an alternative strategy termed cryoprotective dehydration. In this strategy, the difference in water vapor pressure between ice in the soil and the supercooled hemolymph drives a net outflux of water vapor^8,9^. The force of this vapor pressure difference is so large that even a few degrees of supercooling will result in substantial water loss, continuing until the vapor pressure of body fluids equals that of the surrounding ice^8,10^. At this stage, the risk of ice formation in the body has been eliminated, and subzero survival is ensured.

Studies have shown that *M. arctica* on a seasonal timescale efficiently adjusts the melting point of its hemolymph to equal the temperature of its winter habitat^8^. However, in the initial phases of cryoprotective dehydration, melting point depression rates can be slow with the result that hemolymph at this stage is supercooled by a few degrees until water contents have decreased to levels where further evaporative water loss produces higher rates of melting point depression^11^. Even though supercooling is limited to a few °C, and of short duration, physical contact between springtails and ice crystals in the habitat potentially may result in inoculative spread of external ice to hemolymph through hydrophilic surfaces or openings of the animal such as mouth or ventral tube. These considerations prompted us to look for antifreeze proteins (AFPs) which are well-known for their ability to limit ice growth in freeze-avoiding fish and insects^12–14^.

An AFP was previously characterized from a springtail species, *Hypogastrura harveyi*, present in Eastern Ontario^15^. Extracts of active *H. harveyi*, collected from snowbanks in late winter, were able to depress the freezing temperature by 5.8 °C below the melting point. The proteins responsible for this strong thermal hysteresis (TH) activity were unlike any previously described AFPs from fish, insects, plants and microorganisms, being rich in glycine and having tandem tripeptide repeats of Gly-aa2-aa3, where aa2 was frequently also Gly. As predicted by Lin *et al*.^16^, these glycine-rich tracts folded as polyproline type II helices linked by turn regions^17^. The small (6.5 kDa) isoform of *H. harveyi* AFP (*Hh*AFP) had six of these helices forming two sheets secured by intrachain hydrogen bonds in the glycine-rich core, and by two disulfide linkages. A larger isoform (15.7 kDa) was predicted to fold in a similar manner but with 13 polyproline type II helices, again packed into two sheets with a glycine-rich core^18^.

Another reason to probe *M. arctica* for AFPs is the expectation of finding additional novel ice-binding proteins in different Collembola families that might elucidate structure-function relationships in AFPs and the mechanism by which these proteins bind to ice^19^. Four different AFP types have been found in teleost fishes^14^. Several different types have been characterized in insects; and those described to date in plants and microorganisms have added to the remarkable diversity of ice-binding proteins. It is thought that the variety in fish AFPs reflects the relatively recent impact of sea-level glaciation in the Cenozoic era that challenged teleosts to develop ice control at a late stage of their radiation when extant families already existed^20,21^. Collembola are considerably older than teleost fishes, widespread on all continents, including regions where there are sub-zero temperatures, and might therefore yield a similar diversity of AFPs. Indeed, preliminary characterization of *Gomphiocephalus hodgsoni* from Antarctica suggests it contains an AFP with a distinct amino acid composition that is rich is Cys and His^22^.

## Results

### Megaphorura arctica contains a hyperactive AFP

**Figure 1:**
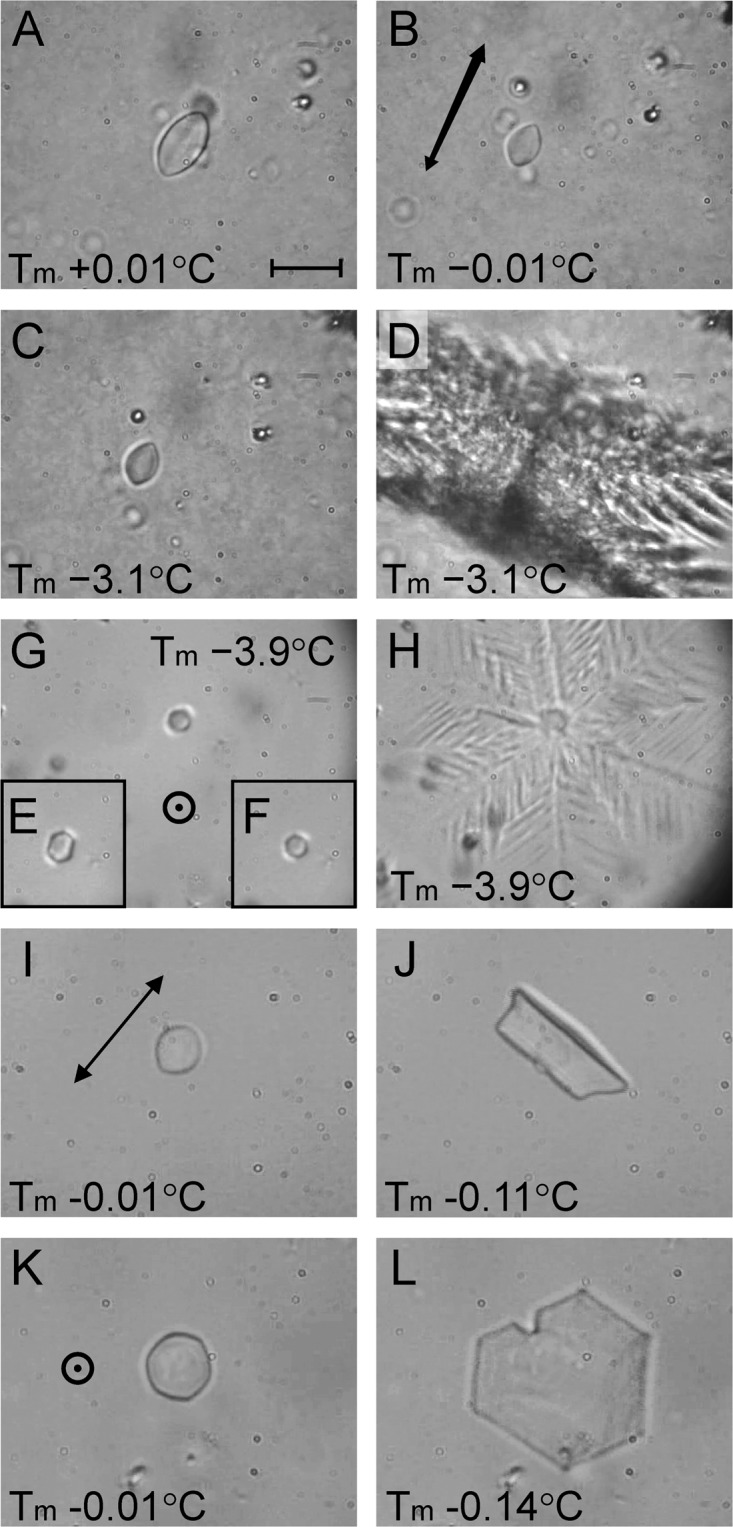
Crystal shaping and thermal hysteresis activity of crude *M. arctica* homogenate (**A**–**D**) and purified native *Ma*AFP (**E**–**H**). (**A**) Crystal shaping attained during melting of monocrystalline ice at 0.01 °C above the melting point (Tm). The scale bar is 20 µm and is the same for all images. (**B**) Final shape of crystal at the start of cooling. The double-headed arrow shows the orientation of the *c*-axis. (**C**) Crystal shape one frame (1/30 s) prior to the burst at 3.1 °C of supercooling. (**D**) First frame once freezing was initiated.(**E**–**H**) Measurements taken on pure *Ma*AFP as described in (**A**–**D**) except that the *c*-axis is perpendicular to the page and the TH was 3.9 °C. The circle with a central dot indicates the ice *c*-axis is normal to the page. (**I**–**L**) Ice crystal shaping attained with diluted purified *Ma*AFP prior to its concentration, with the *c*-axis parallel (**I**–**J**) or perpendicular (**K**–**L**) to the page.

An initial extraction of *M. arctica* (2.3 mg) with buffer (18.4 µL) categorically showed the presence of an AFP in the crude homogenate based on ice crystal shaping and high thermal hysteresis activity (Fig. 1). The ice formed into a distinctive shape with rounded prism surfaces that tapered to points at the basal planes as it was melting (Fig. 1A). This shape was retained unchanged as the temperature was decreased from just under the melting point (Fig. 1B) until 3.1 °C below the melting point (Fig. 1C). When this depressed freezing point was finally exceeded, the ice crystal grew explosively, forming dendritic arms that erupted at angles slightly above and below the plane perpendicular to the *c*-axis linking the crystal tips (Fig. 1D). These characteristics are indicative of hyperactivity and are typical of arthropod AFPs^23,24^.

The larger extraction of freeze-dried *M. arctica* (0.3 g) with buffer (50 mL) was performed to obtain enough AFP for biochemical characterization. The AFP was isolated through four cycles of rotary ice-affinity purification with the emphasis placed on purity rather than yield. Thus, the ice shells were rinsed before being melted and re-extracted; and no back-extractions were performed on the excluded liquid fractions to recover any AFP that was left behind. When the concentrated final ice fraction (500 µL) was tested, it had a thermal hysteresis activity of 3.9 °C, with ice crystal shaping and growth characteristics (Fig. 1E–H) that were similar to those of the original crude extract (Fig. 1A–D). However, in this latter example the crystal orientation happened to be rotated almost 90°, such that the *c*-axis was perpendicular to the image. The six prism surfaces around the circumference of the crystal were evident during melting (Fig. 1E) and the crystal did not visibly change throughout the thermal-hysteresis gap (Fig. 1F–G). When the freezing point was exceeded, the dendritic burst pattern clearly showed the crystal’s hexagonal symmetry with ice growth roughly perpendicular to the *c*-axis (Fig. 1H). When the purified protein was tested at lower concentrations, the crystal still melted into a shape with rounded prism surfaces (Fig. 1I) and the burst was *a*-axial but non-dendritic (Fig. 1J) as the solution was only supercooled by 0.11 °C rather than >3 °C when freezing began. The hexagonal nature of a held crystal and the burst was also evident when the *c*-axis was perpendicular to the page (Fig. 1K,L).

### MaAFP comprises several small isoforms with high-glycine content

**Figure 2:**
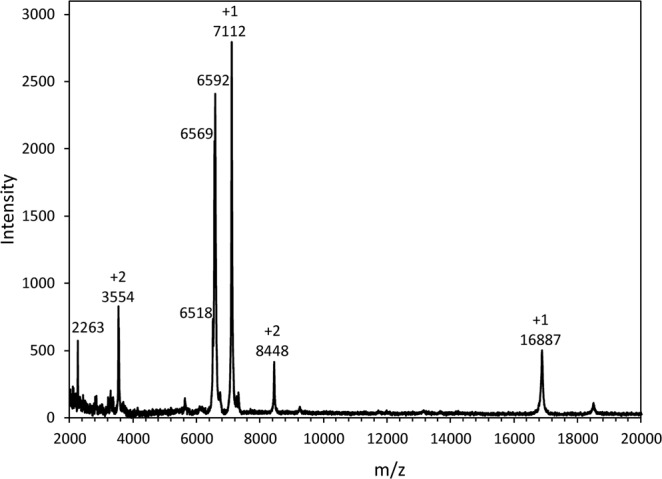
MALDI MS of purified AFPs following four rounds of ice-affinity purification. Peak intensity is plotted against the mass to charge ratio (m/z). Singly (+1) and doubly (+2) charged peaks are indicated.

Analysis of the concentrated pure extract by MALDI mass spectrometry showed the presence of several low molecular weight proteins (Fig. 2). The first large peak included masses of 6518 Da, 6569 Da and 6592 Da, followed by a second large peak of 7112 Da, all with uncertainties of ±5 Da. There was also a smaller peak at 16887 Da ±10 Da. The peaks at 3354 and 8448 m/z are likely doubly-charged versions of the 7112-Da and 16887-Da species respectively. The amino acid composition of the extract (Table 1) was highly enriched in Gly (36 mol%), with Ala (13 mol%) being the next most abundant amino acid. This bias in composition was reminiscent of *H. harveyi* AFP (*Hh*AFP), where the 6.5-kDa isoform had 48 mol% Gly and 14 mol% Ala. The dominance of Gly and Ala also suggested that the several major peaks seen in the MALDI mass spectrum are isoforms of *Ma*AFP rather than impurities. Both purified *Ma*AFP and the *Hh*AFP isoforms had few of the larger amino acids, such as the aliphatic residues (Ile, Leu, Met) and aromatic residues (Phe, Tyr). The somewhat higher combined level of these residues (18%) and lower level of Gly in the ice affinity-purified *Ma*AFP material, compared to the defined sequences, could be due to small amounts of contaminating proteins.

**Table 1:** Molar percentages of amino acids (totaled to 100% with Cys excluded) in various springtail AFPs.

Amino Acid	Purified *Ma*AFPs	*Ma*AFP	*Hh* 6.5 kDa	*Hh* 15.7 kDa
Asx	9	6	9	7
Ser	7	6	4	5
**Gly**	**36**	**49**	**48**	**51**
Thr	4	1	4	3
**Ala**	**13**	**22**	**14**	**16**
Pro	4	8	8	4
Val	5	4	5	2
Lys	3	4	4	2
Arg	2	0	1	1
Other	18	0	3	7

The composition of the purified *Ma*AFPs was determined by amino acid analysis, whereas the compositions of specific isoforms were derived from EST/cDNA sequences (Supplementary Table S1). “Other” includes Met, Leu, Ile, Glx, Tyr, Phe and His. Trp was absent or not detected.

### MaAFP resembles HhAFP in sequence and structure

**Figure 3:**
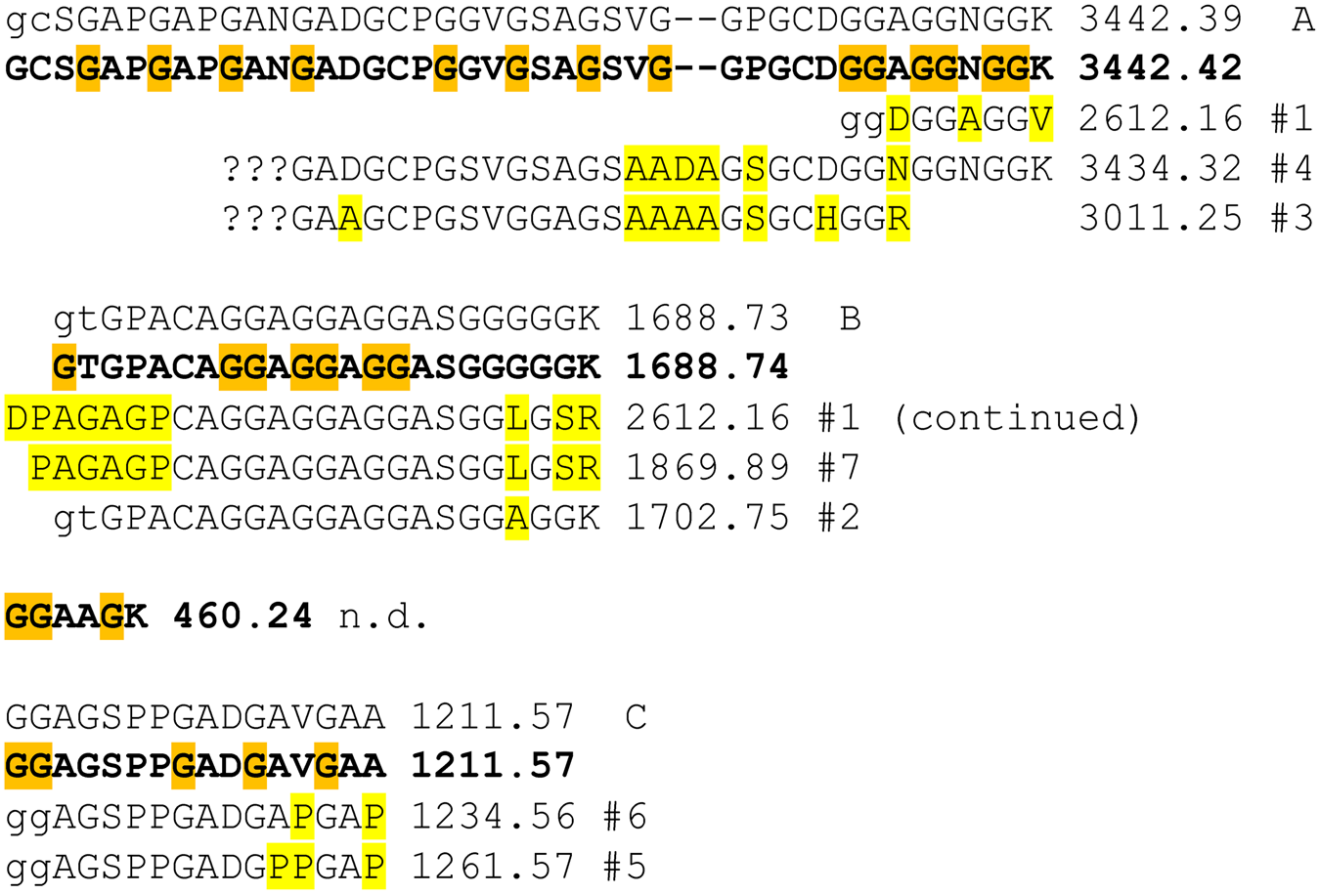
Translation of *Ma*EST EW753059.1 aligned with sequences determined by tandem mass spectrometry sequencing of tryptic fragments from purified *Ma*AFP. The 87-residue open reading frame from the EST (with the signal sequence removed) is shown sequentially in bold as four tryptic fragments of masses 3442.42, 1688.74, 460.24 and 1211.57. Gly residues predicted to form the core are highlighted orange. Above it are the tandem MS/MS sequences that exactly correspond to and support the EST sequence. Below it are homologous fragments producing some of the higher total ion counts (excluding trypsin autolysis fragments) numbered #1 to #7 shown in Supplementary Fig. S1. Differences in these sequenced fragments relative to the bolded EST translation are highlighted yellow. Residues that were inferred from b2 ion masses are in lowercase, and undetermined regions of unknown length are represented by question marks. The predicted (EST) and observed (MS/MS fragment) monoisotopic H^+1^ masses (in Da) are indicated on the right.

The presence of both lysine and arginine in the *Ma*AFP amino acid composition (5 mol% total) encouraged us to digest the protein with trypsin and sequence the resulting peptides (Supplementary Fig. S1) by tandem mass spectrometry (MS/MS). Only one (EW753059) of over 16,000 *M. arctica* expressed sequence tags (ESTs) in the NCBI database^25^ showed significant similarity to any of the MS/MS sequences (Fig. 3). The open reading frame in frame 3 of this DNA sequence (shown in bold) coded for an 87-residue secreted protein (after removal of the signal polypeptide) with a predicted whole mass of 6516.8 Da, which matched the lightest species in the first large peak of the MALDI spectrum (6518 Da). Moreover, the mass and sequence of the three larger (>500 Da) tryptic peptides predicted from the mature sequence (all except GGAAGK) were exactly matched by tryptic MS/MS sequences. In addition, the majority of the polymorphisms observed in the second and fourth fragments, such as A to P, G to L and K to R, would increase the mass of the protein and may explain the peaks at 6569 to 7112 Da. Partial sequences of several other Gly-rich MS/MS sequences from the ice-affinity purified AFPs (not shown) did not align well with EST EW753059, and may correspond to the 16.9-kDa species.

**Figure 4:**
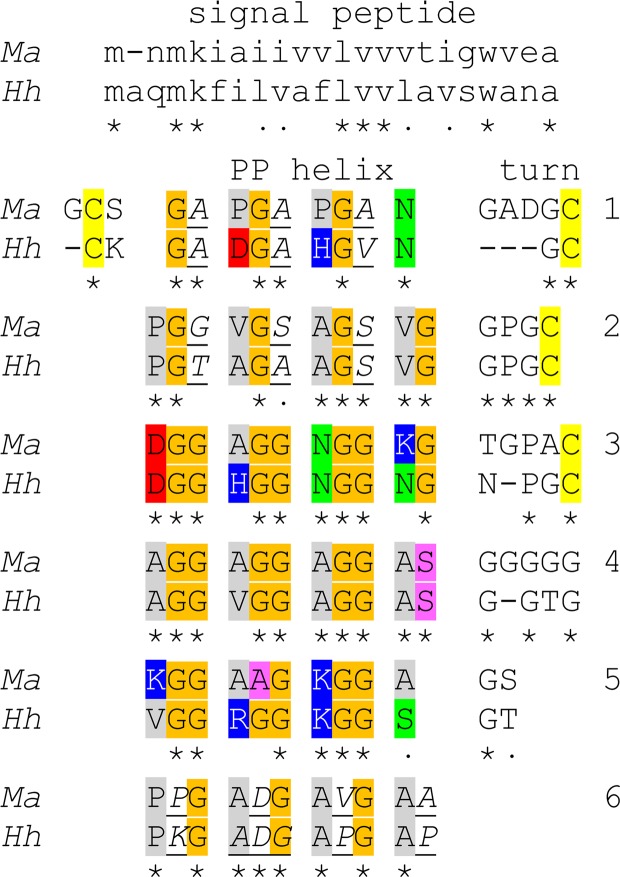
Alignment of *Ma*AFP and *Hh*AFP. Gly residues within the polyproline type II helices that form the core of the proteins are shaded orange. Residues exposed on the ice-binding surface (helices 2, 4, 6) and backside (helices 1, 3, 5) are highlighted grey (hydrophobic), red (acidic), blue (basic) or green (polar). Cys are highlighted yellow; residues with sidechains that are exposed on the sides of the protein are in italics and underlined, and the non-Gly residues within the core are highlighted pink. Identity and similarity are denoted by asterisks and dots, respectively. Signal polypeptides sequences are in lower case at the top.

The sequence of *Ma*AFP contained six short tracts of the tripeptide repeat Gly-aa2-aa3, and in three of these tracts aa2 was also Gly. These sequences and their distribution in the protein are reminiscent of *Hh*AFP. When these two small AFPs were compared they had 62.4% identity, 68.8% similarity and only three gaps in the alignment where *Ma*AFP has additional residues (Fig. 4). The two sequences are aligned to emphasize the Gly-rich tracts linked by more variable turn regions. In both proteins the Gly-rich tracts with the second Gly in the tripeptide repeats were the third, fourth and fifth ones in the sequence. All three gaps in the alignment occur in, or next to, the turns. Also, all four Cys are conserved in their locations.

**Figure 5:**
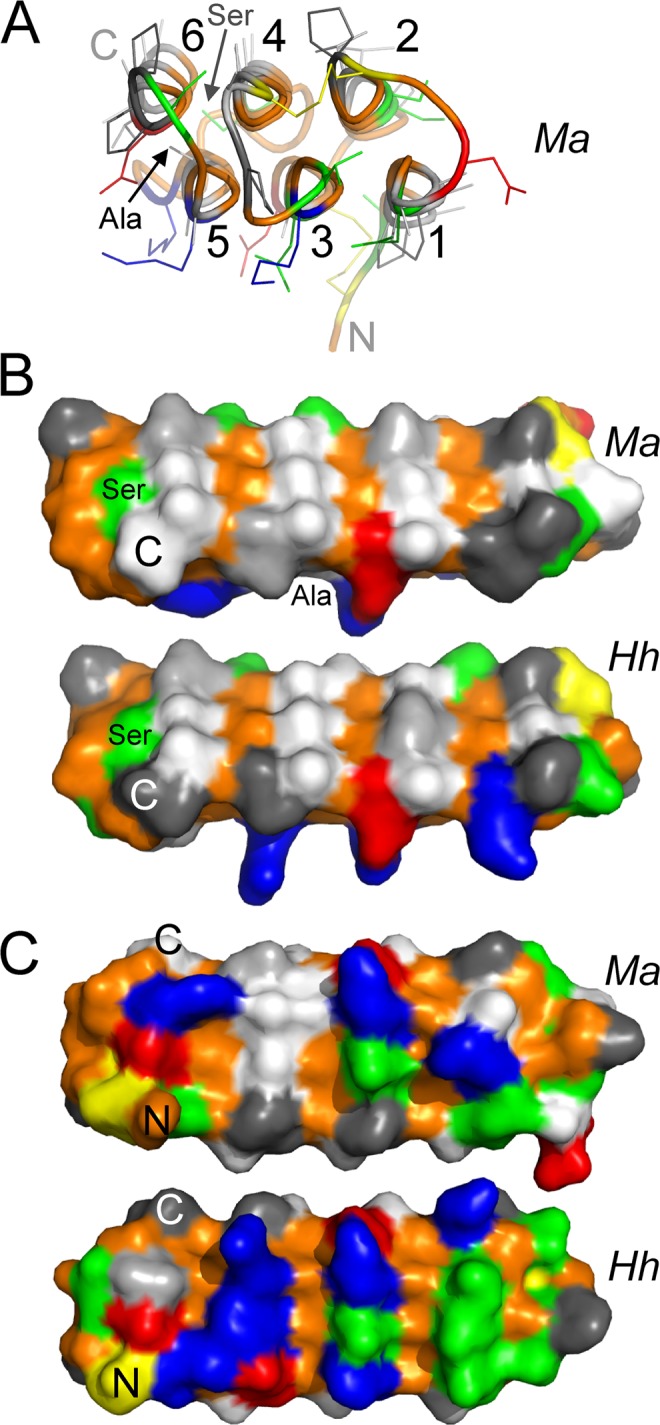
Comparison of the *Ma*AFP model and *Hh*AFP structure. (**A**) End-on view of *Ma*AFP displayed in PyMOL^68^ with the backbone rendered in cartoon mode and sidechains (no H) as sticks, colored as below except Ala were rendered in very light grey. (**B**) Oblique surface rendering of the ice-binding surfaces of *Ma*AFP (top) and *Hh*AFP (bottom). Residues are colored as follows; Ala-white, Val-light grey, Pro-dark grey, Cys-yellow, Gly-orange, basic-blue, acidic-red, polar-green. N- and C-termini are indicated. (**C**) Face-on views of the back surface opposite to the ice-binding surface.

Based on the conserved sequence features mentioned above, it was relatively simple to homology model the three-dimensional fold of *Ma*AFP (Fig. 5). The Phyre 2 web-based system^26^ for protein structure prediction folded the six Gly-rich tracts as polyproline type II helices and packed them into a bundle based on the *Hh*AFP structure. The Phyre2 model had >99% confidence over 93% of the sequence of *Hh*AFP. This model was based on the *Hh*AFP structures solved by Pentelute *et al*.^17^. In this initial model (not shown), helix 4 was rotated relative to the final model with the Ala sidechains directed inwards and the Gly residues surface exposed. This was easily corrected using the program Modeller so that all residues larger than Gly, within the six helices, are now surface exposed (Fig. 5A). The only exceptions to this are the Ser found near the end of helix 4 and the Ala in the second loop of helix 5 (Fig. 4, pink highlighting). The Ser sidechain is easily accommodated as it is adjacent to the C-terminal residue of helix 6. The sidechain of the Ala in helix 5 (which is a Gly in *Hh*AFP) is partially exposed and a small (0.4 Å) shift of the backbone was adequate to prevent steric clashes (Fig. 5A,B). The stability of this model was assessed by molecular dynamics over a 20-ns timespan (Supplementary Fig. S2). The RMSD remained relatively stable with an average deviation of 2.1 Å from the starting point, which was only 0.8 Å higher than that found when the known *Hh*AFP structure was used in the simulation. In both instances, the polyproline helices retained their relative positions and the core Gly residues remained buried.

A comparison of the *Ma*AFP model with the *Hh*AFP structure shows that the two proteins have the same amphipathic nature, where the outer surface formed from the three even-numbered 2^nd^, 4^th^ and 6^th^ helices (Fig. 5A) is relatively hydrophobic because of its high alanine content (Fig. 5B, white residues). This ice-binding site is almost identical between the two proteins and is dominated by outfacing Ala (white), Val (light grey) and Pro (dark grey) residues, where all of the Gly (orange) face inward. The only difference is that one of the Val residues (light grey) is in a different position. The opposite outer surface formed by the odd-numbered helices is more hydrophilic and contains several charged residues like Lys and Asp (Fig. 5C). This is the non-ice-binding site exposed to the bulk solvent when the AFP is bound to the ice surface, and here the residues are not nearly as well conserved. This surface is slightly less hydrophilic in *Ma*AFP as there are several substitutions of charged or polar residues with Ala (white) or Pro (dark grey). The turns at both ends are also somewhat variable, but the two disulfide bonds (Fig. 5B,C) are conserved: linking the start of helix 1 to the end of helix 2, and end of helix 1 to the end of helix 3.

### The untranslated regions (UTRs) of HhAFP and MaAFP are highly divergent

**Figure 6:**
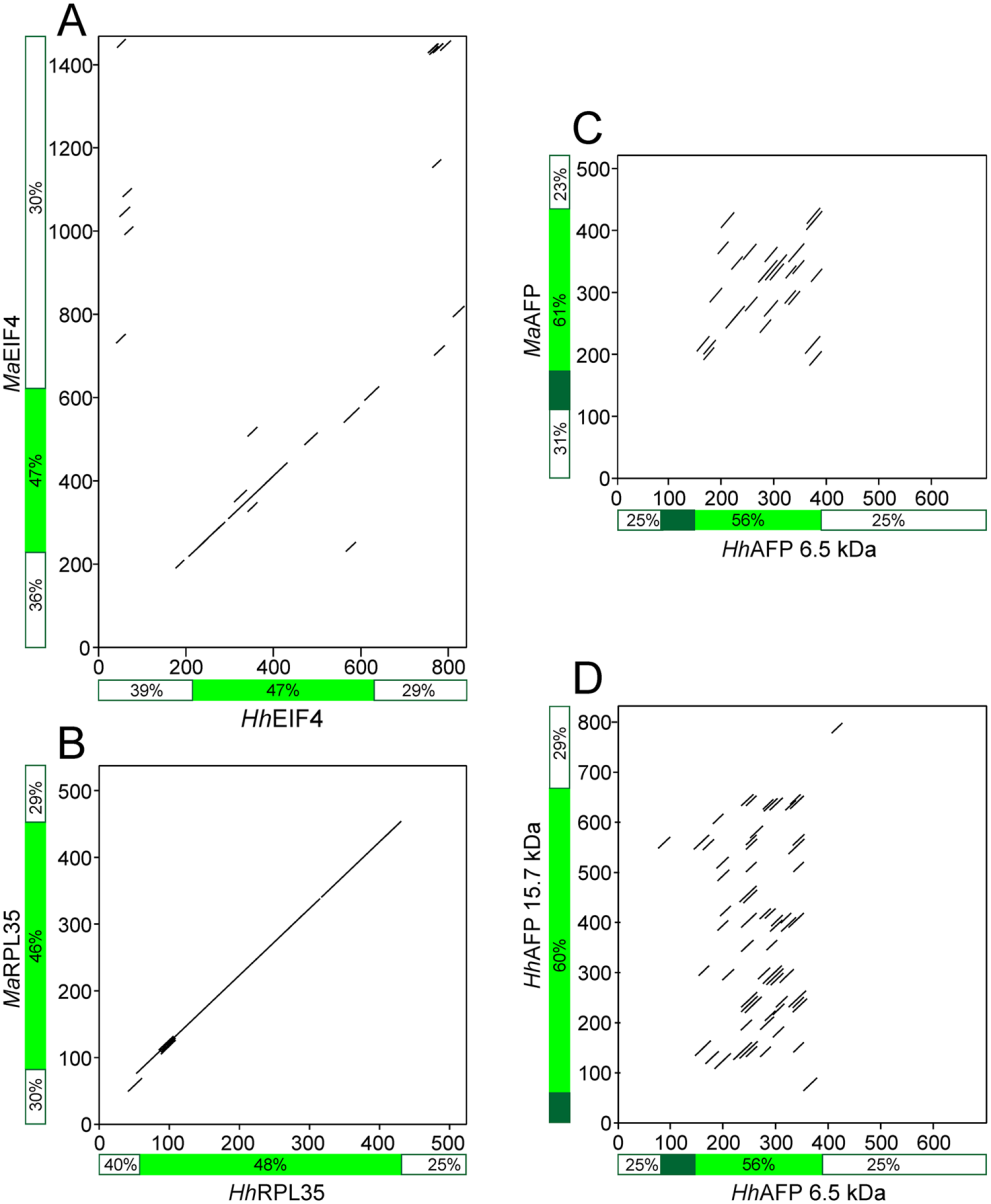
Dot plot comparison of cDNA sequences encoding orthologous proteins (**A**,**B**) or AFP sequences (**C**,**D**) from *M. arctica* and *H. harveyi*. A diagonal line indicates that the two sequences match with a threshold value over 50%, scored across a 20-bp sliding window. All axes are in bp with the 5′ end of each cDNA beginning at zero. The green bars indicate the position of coding sequence with the signal peptide in dark green (if present). The percent GC in each region (5′ UTR, coding region, 3′ UTR) is indicated alongside each axis. Note that the 5′ UTR of *Hh*AFP 15.7 kDa was not sequenced and the green bars do not overlay any diagonal lines. Accession numbers are given in Supplementary Table S1.

To probe the evolutionary relationship between *Hh*AFP and *Ma*AFP we performed dot matrix analyses comparing the cDNAs encoding the AFPs (Fig. 6). For controls we did similar comparisons on two orthologous proteins from these two springtail species. Each 20 bp segment of sequence 1 (sliding in 1 bp increments) was compared to every 20 bp segment of sequence 2. Matches above the threshold are indicated by diagonal lines, which show the relative positions of matches in both sequences.

When cDNAs encoding two well-conserved orthologous proteins (eukaryotic translation initiation factor 4E (EIF4) and ribosomal protein L35 (RPL35)) were compared, the diagonal lines spanning the coding regions indicate that these sequence segments are indeed well conserved (Fig. 6A,B). However, the lack of continuous diagonal lines across the 5′ and 3′ UTRs suggest that the non-coding segments are not conserved. When a similar analysis was done with the AFP sequences of the two species, multiple matches, which appear as numerous parallel diagonal lines, were found between the coding regions of the mature AFPs (Fig. 6C). These diagonals reflect the similarities between the Gly-rich repeats, but the plot lacks the strong continuous diagonal indicative of well-conserved orthologues. In addition, the UTRs and the region encoding the signal peptide are not conserved. A plot comparing the intraspecific large and small AFP-encoding cDNAs of *H. harveyi* showed the same pattern (Fig. 6D). One interesting similarity between the UTRs of all these genes was that they had a reduced GC content (23 to 40%) relative to the coding sequences (46 to 61%). The scattered matches between the 3′ UTRs of *Ma*- and *Hh*-EIF4 (Fig. 6A) correspond primarily to A-rich tracts within these AT-rich sequences and are not indicative of homology.

## Discussion

Examples of highly potent AFPs are mostly found in freeze-avoiding insects with high supercooling capacity^27–30^. For instance, AFP-mediated freezing point depression in the spruce budworm (*Choristoneura fumiferana*) of 6 °C is an important component in overwintering of first-instar larvae remaining unfrozen and viable in the boreal forest at temperatures of −20 to −30 °C^28^. Despite *M. arctica*’s reliance on cryoprotective dehydration, and not deep supercooling, as its main strategy for avoiding freezing, these springtails also possess a potent AFP that complements the set of adaptive traits for winter survival in this species.

Due to logistic limitations we were not able to measure antifreeze hysteresis activity of hemolymph samples from live specimens in the field, and we therefore do not have a direct determination of the amount of freezing point depression *Ma*AFP can afford its host. However, since we know the dry weight of the animal sample and how much buffer was used for the crude extract measurements, we can make an estimate of this. We made an extract from 2.3 mg dry *M. arctica* tissue in 18.4 µL of buffer, equivalent to a water content of ca. 7.8 mg water mg^−1^ dry tissue, which is at least twice the normal water content of fully hydrated *M. arctica*^31,32^. It is therefore likely that the freezing point depression provided by *Ma*AFP *in vivo* is similar or perhaps even higher than the 3.1 °C measured for the crude extract.

Since cryoprotective dehydration can sometimes be a slow process some supercooling capacity (typically 2–4 °C) is needed in the initial phases of dehydration, especially if the cooling rate is high^8,31,33^. At this stage, there is a risk that external ice will spread to the animal causing inoculative freezing. Until now, we have been missing a good explanation how inoculative freezing is avoided in frozen soil, but the discovery of a potent AFP in *M. arctica* closes this gap in knowledge. Thus, the freezing point depression that we estimated to ca. 3 °C seems to meet with the needs outlined above. Studies of the exact location of *Ma*AFP in the animal would further explain the ability to avoid inoculative freezing. AFP can be expressed in various tissues of insects such as the midgut, hemolymph and epidermis^34,35^. Expression of AFP in epidermis cells could be particularly relevant for inoculative freezing in *M. arctica* and other springtails using cryoprotective dehydration^34^.

Inoculative freezing may be a significant cause of lethality for springtails and many other soil living invertebrates that do not tolerate freezing of their body fluids. For example, Sømme and Conradi-Larsen (1977) observed that cooling the springtail *Tetracanthella wahlgreni* to −3 °C in close contact with ice caused inoculative freezing and death even though supercooling points of this species ranged between −15 and −25 °C if specimens were not in contact with external ice^36^. Since contact with ice is decisive, the risk of inoculative freezing is highest when soil moisture content is high^37,38^. Interestingly, several hygrophilic soil invertebrates (e.g. nematodes, enchytraeids and midges) survive sub-zero temperatures by freeze-tolerance, or can switch between cryoprotective dehydration and freeze-tolerance depending on the moisture conditions of their overwintering habitat^39–41^. This dual strategy is consistent with the fact that both freeze-tolerance and cryoprotective dehydration involves tolerance of cellular dehydration and the physiological adaptations connected to this^11^.

*Ma*AFP has the attributes of a hyperactive antifreeze protein in having high thermal hysteresis activity at low protein concentrations, with evidence of AFP binding to the basal plane. This is typical of arthropod AFPs, which have generally been selected for higher freezing point depression activity than the moderately active AFPs in fish, which never face temperatures lower than −1.9 °C^23,24^. *Ma*AFP is made up of what appear to be several isoforms. Multiple AFP isoforms are common in fish^42–45^ and arthropods^35,46,47^. They arise from gene duplication followed by divergence. Since freezing point depression is a function of AFP concentration, AFP-producing organisms seem to be under selective pressure to increase the amount of circulating AFP by gene amplification^42,48^. For AFPs made from repetitive sequences it is easy to generate length variation by expansion and/or contraction of the repeats. Examples of this can be seen in AFPs from the beetles *Tenebrio molitor* and *Dendroides canadensis* where the repeating unit is a 12-residue coil in a beta-solenoid structure. Common isoforms have seven coils but some have eight^46,49^. Another example of length variation is in the moth, *Choristoneura fumiferana*, where one isoform has two extra 15-residue coils in its beta-solenoid structure^50^. Interestingly, these larger isoforms are more active than the shorter ones because the ice-binding site is increased in area. Thus, there may also be selective pressure to increase isoform size.

Lengthening of the polyproline type II coils in *Hh*AFP and *Ma*AFP seems feasible too, since they are based on Gly-aa2-aa3 tandem tripeptide repeats. However, unlike the single solenoid structures of the beetle and moth AFPs, the polyproline type II coils must pack into a higher order tertiary structure where the coils are bundled together to form a Gly-rich core. Thus, the expansion of a single coil would potentially destabilize the overall structure. Instead, an increase in isoform size in *Hh*AFP seems to have been accommodated by an increase in the number of coils from 6 to 13, and with it again a considerable increase in antifreeze activity^18^.

Despite the high sequence identity between *Ma*AFP and *Hh*AFP it is not possible to unequivocally say they are descended from a common ancestor sequence. There are well-documented examples of simple repetitive AFPs that have independently originated from different progenitors. For example, the antifreeze glycoproteins (AFGPs) of Antarctic Notothenioids and Arctic cods are made up of a tandemly repeated Ala-Ala-Thr tripeptide with a disaccharide O-linked to the Thr^44,45^. Although these AFGPs are virtually indistinguishable and share almost complete sequence identity as a set of isoforms of different lengths derived from polyprotein precursors, they are not homologs. The Notothenioid AFGPs have arisen from an intronic sequence from the gene for the pancreatic enzyme, trypsinogen^51,52^. AFGPs of the northern cods, however, are derived from a different DNA sequence^53^. Type I AFPs are another example of convergent evolution to a structural and functional commonality. These Ala-rich, alpha-helical AFPs, with an underlying 11-amino-acid repeat, have independently arisen at least four times in different branches of teleost fishes from unknown precursor sequences^54^. Since they are 60–65% Ala they all have >25% sequence identity, which is the measure applied to recognize homology. Clearly this criterion is invalid when dealing with proteins that have such a biased amino acid composition.

The comparison between *Ma*AFP and *Hh*AFP is more complex because, unlike the AFGP and type AFP I examples above, these AFPs have a higher order of protein structure where six helices of similar length pack together to form a Gly-rich core. In addition, there are four Cys, whose positions are conserved such that the two disulfide bonds they form to help secure the helix bundle structure are in the same places in both proteins. These structural features argue for homology. A comparison of the 5′- and 3′-untranslated regions shows no sign of sequence identity, which could argue against homology. One possible explanation for these confounding results is that *Ma*AFP and *Hh*AFP are ancient homologs that have survived from an earlier ice age, perhaps even before these two species diverged. Collembola originated at least 400 Ma and would have encountered the ice ages of the Permian period ~300 Ma^55–58^. If so, some of the extant springtails may have derived their AFPs from this period. Those that retained their AFP genes during the 200+ million years between the Permian and Cenozoic ice ages would have been well equipped to survive in present sub-zero conditions. This hypothesis might explain why the UTRs of the *Hh*AFP large and small isoforms also appear unrelated, as do the UTRs of *Ma*AFP and *Hh*AFP. If the isoforms predate speciation and date back to the Permian period, regions of the gene not under selective pressure would have had ~300 million years to drift. One way to test this hypothesis would be to sample additional freeze-resistant springtail species for the type of AFPs they might possess to see if they too have high-Gly, polyproline type II helical bundles suggestive of an early origin.

## Materials and Methods

### Collection and storage of springtails

Adult *M. arctica* were collected in August 2017, on a beach near Sauðárkrókur, north-west Iceland, and brought back to the laboratory in Silkeborg, Denmark. The animals were kept at 5 °C in 200-mL beakers on a substrate of decomposing brown algae. In order to acclimate springtails to winter conditions, they were kept in darkness at 5 °C for three months followed by about two months at 1.5 °C. At this point, ~1,500 adult animals were taken and freeze dried for two days to complete dryness. The dried animals were stored in Eppendorf vials at −80 °C until they were shipped by courier to Queen’s University, Canada.

### Antifreeze activity measurements

Antifreeze activity, referred to here as thermal hysteresis (TH), was measured in a nanolitre osmometer^59^ as the depression of the freezing point (°C) below the melting temperature. To establish the presence or absence of TH, a soluble extract was prepared from freeze-dried *M. arctica* (2.3 mg). The dried tissue was mixed with 18.4 µL of homogenization buffer (50 mM Tris-HCl (pH 7.8), 100 mM NaCl, 2 mM phenylthiocarbamide, 1x cOmplete, EDTA-free protease inhibitor tablet (Roche, Mississauga, Canada)) and homogenized by hand, using a pellet pestle (Sigma, Burlington, MA) in a 1.5-mL tube. The homogenate was centrifuged at 16,000 × g for 15 min and the supernatant tested for antifreeze activity.

### Ice-affinity purification of AFP

For a larger-scale extraction, freeze-dried *M. arctica* (0.30 g) were homogenized at 4 °C in 50 mL of the above buffer containing 1 mM EDTA. The homogenate was centrifuged at 31,000 × g for 30 min, and the supernatant was filtered through glass wool to remove lipid. AFP was extracted from the filtered supernatant (<50 mL) using four cycles of rotary ice affinity purification^60,61^, but scaled down in size from the previously reported protocol. Thus, the starting ice shells contained 10–15 mL of ddH_2_O in a 250-mL round bottom flask. The rotating ice-shells were cooled in an ethylene glycol bath for 1.5–2 h starting at −1.5 °C and decreased by 0.2 °C every 0.5 h until ~25 mL of liquid remained in the flask. Once the liquid was ~50% incorporated into the ice-shell the 250 mL flask was removed from the apparatus, the liquid was poured out and diluted to 50 mL with ddH_2_O, and the ice-shell was quickly rinsed with 5 mL of homogenizing buffer. The ice shell was melted with 5 mL of 10× homogenate buffer containing 10 mM PTU and 10 mM PMSF then diluted to 50 mL with ddH_2_O. This process was repeated for four rounds of ice-affinity purification. The final melted ice sample was concentrated using an Amicon Ultracel 3 K Centrifugation filter spun in a refrigerated Sorvall ST16R centrifuge at 4000 rpm until a volume of ~500 𝜇L was reached.

### Amino acid analysis

Amino acid composition analysis was performed at the SickKids Proteomics, Analytics, Robotics & Chemical Biology Centre (SPARC) BioCentre as previously described^61^.

### Mass spectrometry

MALDI mass spectrometry was performed using α-cyano-4-hydroxycinnamic acid matrix on dried droplets with a SCIEX Voyager DE Pro in Linear mode by the Queen’s Protein Function Discovery Facility. Tandem mass spectrometry protein sequencing of tryptic fragments separated by high-performance liquid chromatography (LC-MS/MS) was done using ESI (nanospray) with an Orbitrap (FT-ICR) by SPARC BioCentre Molecular Analysis (The Hospital for Sick Children, Toronto, Canada).

### Transcriptome data analysis

*H. harveyi* AFP sequences (ABB03725.1 and ADI39904.1) were used as query sequences in BLAST searches (NCBI, with compositional adjustments and low-complexity filters off) of *M. arctica* sequences^62^. Additionally, all 16379 *M. arctica* ESTs were downloaded from NCBI, and translations of all six reading frames^63^ were scanned for sequence strings identified by LC-MS/MS. To find control sequences for dot blot comparisons, previously sequenced *H. harveyi* ESTs for nuclear genes were searched by BLAST^64^ for two examples that encoded homologous sequences in the *M. arctica* EST database. These were then assembled into contigs using the Cap3 Sequence Assembly Program^65^. Dotmatcher http://www.bioinformatics.nl/cgi-bin/emboss/dotmatcher, written by Ian Longden, formerly at the Sanger Institute, Wellcome Trust Genome Campus, Hinxton, Cambridge, CB10 1SA, UK, was used to compare dot plots of the two nuclear gene cDNAs with complete coding sequences, 5′ UTRs ≥ 60 bp and complete 3′ UTRs, as well as cDNAs encoding the AFPs.

### Molecular modeling

The *Ma*AFP sequence was submitted to Phyre2^26^ using both normal and intensive mode. An alignment sequence of *Ma*AFP against *Hh*AFP was inputted into MODELLER which performed homology modeling to produce the refined model of *Ma*AFP^66^. GROMACS was used with both the refined model of *Ma*AFP and the crystallographic structure of *Hh*AFP^67^. The structures were energy minimized after being solvated in a box of water, after which 0.1 ns position-restrained molecular dynamics runs at constant-volume and constant-pressure were done. These were followed by unrestrained molecular dynamics at a temperature of 277 K for 20 ns where temperature and pressure were maintained with the v-rescale and Parrinello–Rahman protocols, respectively.

## Supplementary information

Supplementary information.

## Data Availability

The datasets generated during and/or analyzed during the current study are available from the corresponding author on reasonable request.
