# Supplementary material for: Antifreeze protein complements cryoprotective dehydration in the freeze-avoiding springtail Megaphorura arctica
Source: Sci Rep. 2020 Feb 20;10:3047. doi: 10.1038/s41598-020-60060-z (PMC7033094; doi:10.1038/s41598-020-60060-z)
Supplement: Supplementary file 1 — Supplementary information. [file 41598_2020_60060_MOESM1_ESM.pdf]

Antifreeze protein complements cryoprotective dehydration in the freeze-avoiding springtail *Megaphorura arctica*

Laurie A. Graham[1], Marie E. Boddington[1], Martin Holmstrup[2,3] and Peter L. Davies[1*]

[1] Department of Biomedical and Molecular Sciences, Queen's University, Kingston, ON, Canada

[2] Section of Terrestrial Ecology, Department of Bioscience, Aarhus University, Vejlsøvej 25, 8600 Silkeborg, Denmark.

[3] Arctic Research Center, Aarhus University, Ny Munkegade 114, 8000 Aarhus C, Denmark.

*Corresponding author: Peter L. Davies. Email: daviesp@queensu.ca

Supplementary Materials

Supplementary Table S1. GenBank accession numbers of sequences used to generate the dot plots in Fig. 6. Contigs were generated when multiple ESTs were available.

*Ma*EIF4	*Ma*RPL35)	*Hh*AFP 6.5 kDa
EW747994.1	EW749187.1	DQ177322.1
EW748704.1	EW758331.1	
EW748778.1		
EW749550.1		
EW751327.1		
EW752258.1		
EW753354.1		
EW755035.1		
EW755648.1		
EW757273.1		
EW758575.1		
EW758606.1		
EW758680.1		
EW759859.1		

*Ma*AFP	*Hh*AFP 15.7 kDa
EW753059.1	GU169329.1

*Hh*EIF4	*Hh*RPL35
MN649842	MN649843

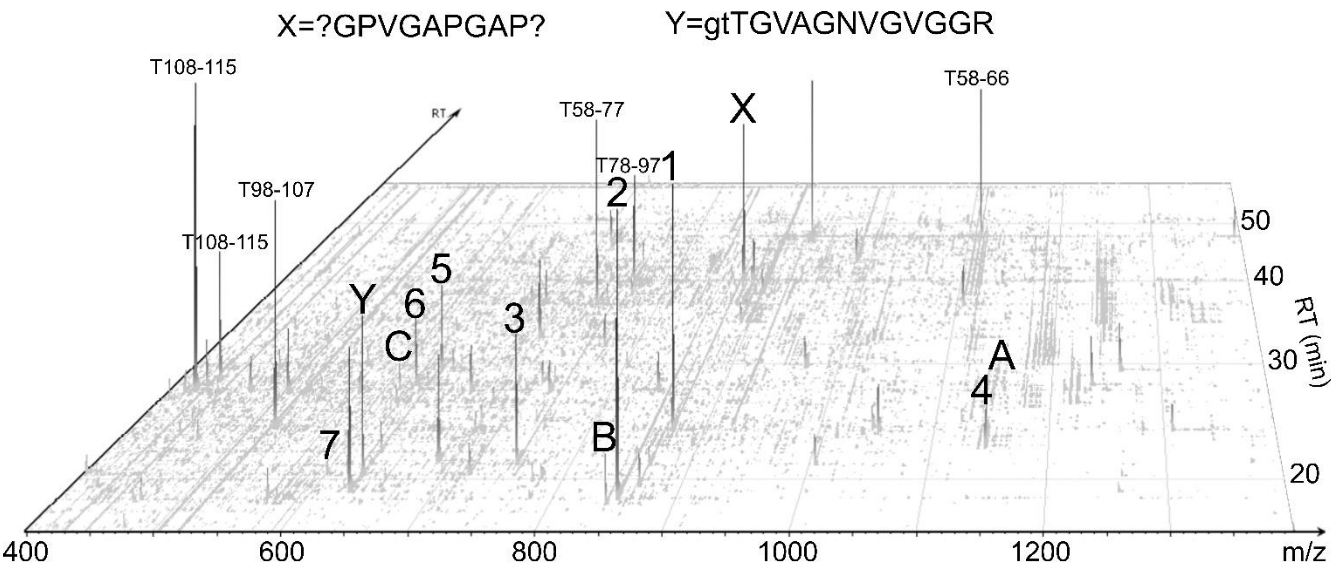

Supplementary Figure S1. LC-MS/MS heatmap of tryptic fragments from purified *Ma*AFP. Peak height (y-axis) represents ion intensity with m/z on the x-axis and retention time (RT) on the z-axis. Fragments are labelled above each peak as in Fig. 3, with A-C corresponding to the EST sequence, 1-7 corresponding to polymorphic fragments, X and Y corresponding to two fragments that cannot be unambiguously aligned to the EST and T plus residue numbers corresponding to tryptic autolysis fragments. The heatmap was generated using Peaks V8.5. (Ma B, Zhang K, Hendrie C, Liang C, Li M, Doherty-Kirby A, Lajoie G. PEAKS: powerful software for peptide de novo sequencing by tandem mass spectrometry. Rapid Commun Mass Spectrom. 2003;17(20):2337-42. PubMed PMID: 14558135).

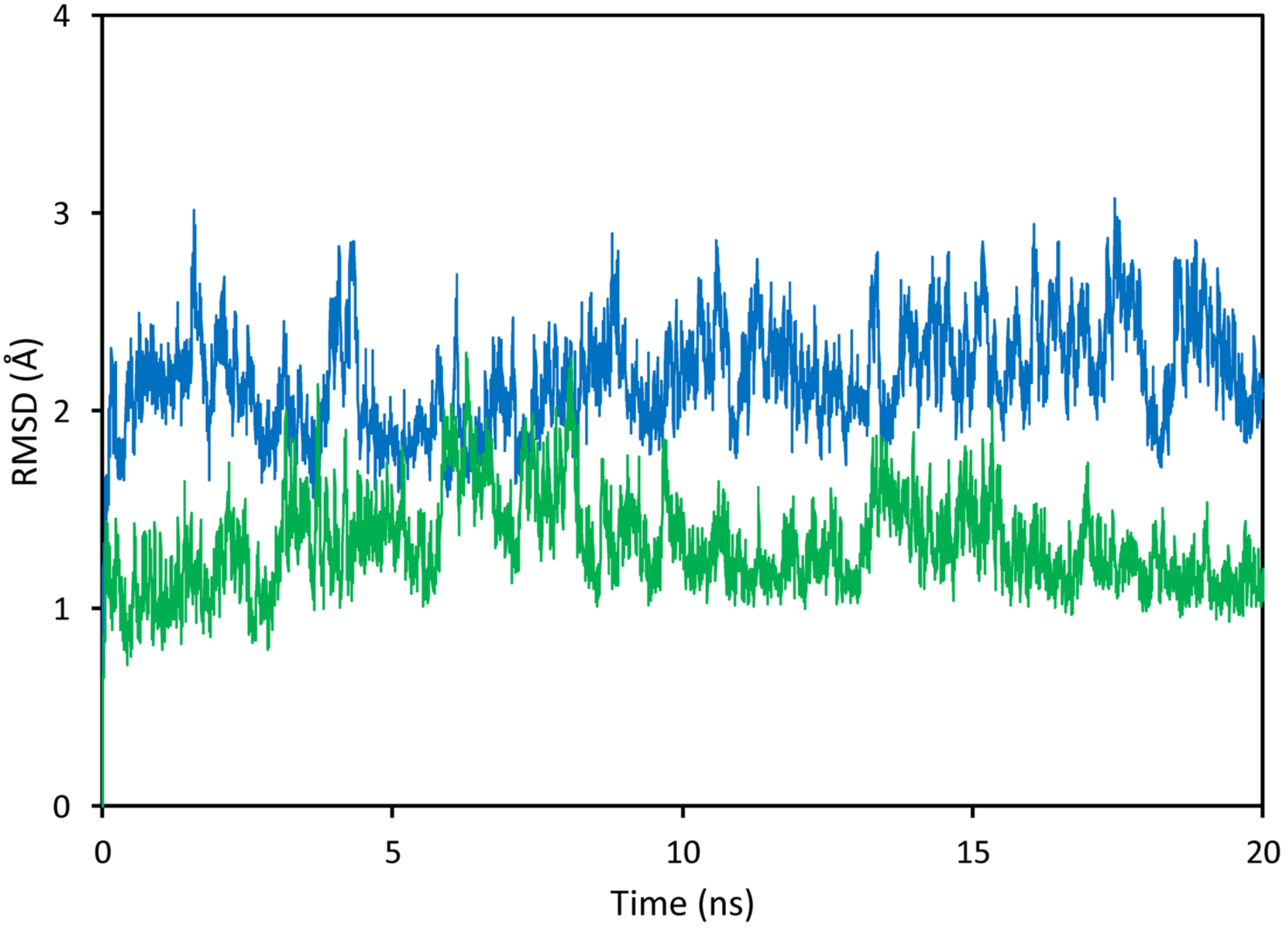

Supplementary Figure S2. Molecular dynamics analyses. Root mean squared deviation (RMSD) of an *Ma*AFP model (blue) compared to that of the known structure of *Hh*AFP (green, PDB 2pne) during a molecular dynamics simulation at 4 °C which included all but the hydrogen atoms.
